# Evidence for chronic inflammation in cats with cardiomyopathies

**DOI:** 10.1177/1098612X251385885

**Published:** 2025-09-25

**Authors:** Martina Kroficˇ Žel, Kun-Ho Song, Alenka Nemec Svete, Aleksandra Domanjko Petricˇ

**Affiliations:** 1 Veterinary Faculty, Small Animal Clinic, University of Ljubljana, Ljubljana, Slovenia; 2 College of Veterinary Medicine, Chungnam National University, South Korea

**Keywords:** Chronic inflammation, neutrophil:lymphocyte ratio, monocyte:lymphocyte ratio, systemic inflammation response index, cardiomyopathies, pleural effusion, pulmonary oedema, ACVIM B, ACVIM C

## Abstract

**Objectives:**

The study aimed to investigate the extent and type of inflammation using the complete blood count (CBC) and selected CBC-derived inflammatory markers (neutrophil:lymphocyte ratio [NLR], monocyte:lymphocyte ratio [MLR] and systemic inflammation response index [SIRI]) in cats with cardiomyopathy stages American College of Veterinary Internal Medicine (ACVIM) B and ACVIM C vs healthy cats. The second aim was to find any differences in CBC and CBC-derived inflammatory markers between cardiogenic pleural effusion and cardiogenic pulmonary oedema.

**Methods:**

For comparison between the control, ACVIM B and ACVIM C groups, one-way analysis of covariance (ANCOVA) or Quade’s non-parametric ANCOVA, with age included as a covariate, was used. The independent *t*-test or Mann–Whitney test was used for comparison of data between cats with pulmonary oedema and those with pleural effusion. A value of *P* ⩽0.05 was considered significant.

**Results:**

A total of 66 cats with cardiomyopathy (33 ACVIM B and 33 ACVIM C) and 24 healthy cats were included in the study. Cats in the ACVIM C group had a significantly higher white blood cell concentration than those in the ACVIM B control groups. Cats in the ACVIM C group had significantly higher neutrophil concentration, NLR, MLR and SIRI than healthy cats. Cats in the ACVIM B group had a significantly higher NLR and SIRI than healthy cats. Cats with pulmonary oedema and cats with pleural effusion did not differ significantly in any of the investigated CBC and selected CBC-derived inflammatory markers.

**Conclusions and relevance:**

These results support the presence of inflammation in feline cardiomyopathies, particularly in the ACVIM C stage. With the parameters used, no differences in the extent or type of inflammation between cardiogenic pulmonary oedema and pleural effusion was demonstrable.

## Introduction

Hypertrophic cardiomyopathy (HCM) is the cause of death in approximately 25% of cats.^1–4^ Its prevalence increases with age and affects up to 15% of apparently healthy adult cats.^5^ It is suggested that myocardial remodelling in feline HCM might be macrophage-driven, which is associated with the upregulation of profibrotic genes, formation of new vessels, proliferation of fibroblasts and deposition of collagen.^6,7^ Inflammatory cell infiltrates and increased myocardial collagen have been observed in cats with mild preclinical HCM.^8^ Similarly, inflammatory cytokines are recognised to be associated with human^9^ and feline^7^ HCM.

In human patients, low-grade chronic inflammation has been associated with the pathogenesis of HCM, congestive heart failure (CHF) and acute heart failure.^9–11^ Inflammatory biomarkers – such as C-reactive protein (CRP), complete blood count (CBC) parameters, platelet:lymphocyte ratio (PLR), lymphocyte:monocyte ratio (LMR), systemic immune-inflammatory index (SII) and systemic inflammation response index (SIRI) – were shown to be associated with the risk of all-cause mortality.^10^ SII is defined as the product of platelet and neutrophil concentrations divided by lymphocyte concentration, while SIRI is a newly used marker calculated as the product of neutrophil and monocyte concentrations divided by lymphocyte concentration. SII, which includes platelets, is linked to vascular and acute systemic inflammation, especially in thrombosis, infections and malignancies.^12,13^ In contrast, SIRI incorporates monocytes and may better reflect chronic or adaptive immune responses.^14^ Both markers reflect different aspects of inflammation than CRP.^15^

Inflammation has also been found in dogs with advanced stage heart failure.^16–21^ It was found that higher neutrophil:lymphocyte ratio (NLR), monocyte:lymphocyte ratio (MLR) and PLR were linked to the severity of the disease and predicted shorter survival times in dogs with myxomatous mitral valve disease (MMVD).^22^ Moreover, NLR and MLR were proposed as adjunctive indicators of diagnostic and clinical treatment response in dogs with CHF.^23^

NLR and PLR have prognostic value in various feline diseases, including cardiorespiratory diseases.^24^ Recently, NLR was significantly associated with echocardiographic measures of left atrial size, left auricular function, the presence of left atrial spontaneous echo contrast and thrombus formation in feline HCM.^25^ The most common clinical presentation of feline cardiomyopathies is left heart failure, which may result in pulmonary oedema and/or pleural effusion.^26^

Except for NLR, CBC-derived inflammatory markers have not been studied in cats with cardiomyopathy. The aim of the present retrospective study was to assess the extent and type of inflammation using CBC parameters and selected CBC-derived inflammatory markers (NLR, MLR, SIRI) in cats with cardiomyopathy (American College of Veterinary Internal Medicine [ACVIM] stages B and C) vs healthy cats and to compare these markers between cats with cardiogenic pleural effusion and pulmonary oedema.

We hypothesise that systemic inflammation is greater in cats with advanced cardiomyopathy and may vary depending on the type of congestive manifestation, such as pulmonary oedema vs pleural effusion.

## Materials and methods

We retrospectively analysed data from echocardiographic examinations and haematological and biochemical analyses of client-owned cats examined at the Cardiology Department of the Small Animal Clinic of the Veterinary Faculty between 2015 and 2024. Cats diagnosed with HCM or restrictive cardiomyopathy (RCM) were included if CBC was available. Cats with identified concurrent diseases, such as acute systemic inflammation from other causes, liver disease, pyothorax or cancer, were excluded from the study.

Cats were assessed and staged according to the ACVIM consensus statement guidelines for the classification, diagnosis and management of feline cardiomyopathy.^27^

In cats diagnosed with HCM or RCM and CHF, radiography was performed in the right lateral and dorsoventral positions (Iconos R200; Siemens and Luminos Lotus Max; Siemens). Pulmonary oedema was diagnosed based on the enlargement of lobar pulmonary veins and the presence of a patchy interstitial and alveolar pattern in the lung parenchyma.

Pleural effusion was diagnosed using thoracic radiography and/or ultrasonography.

Cats with cardiomyopathies were divided into two groups: ACVIM C and ACVIM B. Cats with CHF (ACVIM C) were further divided into pulmonary oedema or pleural effusion groups.

Furthermore, a control group of healthy cats with similar age and weight was included. To be classified as healthy, the physical examination and haematological, biochemical and urinalysis results had to be within the reference interval. Moreover, the cats had to be feline leukaemia virus (FeLV)/feline immunodeficiency virus (FIV) negative, with no audible heart murmur on auscultation.

All procedures complied with the relevant governmental regulations regarding animal welfare.

Blood samples were collected from the jugular vein for the determination of CBC and serum biochemical profiles (urea, creatinine, alanine aminotransferase, alkaline phosphatase, total proteins, albumins, total calcium, inorganic phosphate). A rapid immunoassay for simultaneous detection (SNAP FIV/FeLV Combo Test; IDEXX) of FeLV antigen detection and FIV-specific antibody was also performed.

Biochemical profiles were determined using an RX Daytona automated biochemistry analyser (Randox), while haematological parameters were measured with an ADVIA 120 automated laser haematology analyser (Siemens) using species-specific software.

In cases where a platelet clumps flag (PLT-CLM) was reported in the ADVIA 120 haematology analyser output, the cats with such results were excluded from the study. The exclusion was necessary not only because clumps artifactually lower platelet counts, but also because platelet clumps can distort other haematologic measurements leading to erroneous white blood cell count (WBC) and WBC differential results, as well as red blood cell indices due to altered light-scattering properties during analysis.^28–30^

CBC-derived inflammatory markers were calculated from numerical values of the WBC differential.

Serum total T4 concentration was measured using a MiniVidas analyser (bioMérieux).

Statistical analyses were performed using commercial software (SPSS version 28; IBM). The Shapiro–Wilk test was performed to determine the distribution of data. Descriptive statistics summarised demographic and laboratory data of individual groups of cats. Depending on the distribution, either parametric (normally distributed data) or non-parametric (non-normally distributed data) statistical tests were applied. The parametric independent *t*-test or non-parametric Mann–Whitney test were used to compare data between cats with pulmonary oedema and those with pleural effusion and between ACVIM B1 and ACVIM B2 groups.

To compare data among the control, ACVIM B and ACVIM C groups, we used parametric one-way analysis of covariance (ANCOVA) or Quade’s non-parametric ANCOVA, with age included as a covariate to control for its potential confounding effect. Age and weight were compared across the control, ACVIM B and ACVIM C groups using the non-parametric Kruskal–Wallis test, followed by Dunn’s post-hoc test with Bonferroni correction. Reported *P* values were adjusted for multiple comparisons and obtained directly from SPSS output.

In addition, we performed a post-hoc power analysis. The observed power values were obtained directly from the ANCOVA and Quade’s ANCOVA output tables generated by the software.

A value of *P* ⩽0.05 was considered significant. Normally distributed data are presented as mean ± SD, whereas non-normally distributed data are presented as the median and interquartile range (IQR; 25th–75th percentiles) or represented in box plots.

## Results

A total of 115 records of cats diagnosed with HCM or RCM were evaluated. In total, 49 cases were excluded because of concurrent systemic inflammatory diseases or the absence of CBC data. Ultimately, 33 cats in ACVIM B (22 ACVIM B1 and 11 B2) and 33 cats with CHF (ACVIM C) were included. The ACVIM B1 and B2 subgroups did not differ significantly in any of the investigated CBC parameters and CBC-derived inflammatory markers; therefore, the cats in these two groups were not statistically evaluated separately.

Three cats in ACVIM C exhibited neither pulmonary oedema nor pleural effusion at the time of inclusion and were therefore not included in the statistical comparison of the pulmonary oedema and pleural effusion groups. There were more male than female cats in both the ACVIM B and C groups (Table 1).

**Table 1:** Signalment and renal parameters of the cats included in the control, American College of Veterinary Internal Medicine (ACVIM) B and ACVIM C groups

Parameter	Control group	ACVIM B	ACVIM C	*P* value*
Number of cats	24	33	33	–
Sex (M/F)	14/10	22/11	23/10	–
Age	7 years (6 to 10 years and 9 months)	12 years (5 years and 9 months to 14 years and 9 months)	10 years (7–13 years)	0.117^†^
Body weight (kg)	4.95 (3.84–5.75)	4.73 (3.40–7.31)	4.52 (3.50–7.05)	0.985^†^
Breed				
Domestic shorthair	23	25	28	–
Maine Coon	–	5	1	–
Ragdoll	–	1	–	–
Birman	1	–	1	–
British Shorthair	–	–	2	–
Sphynx	–	1	1	–
Exotic Shorthair	–	1	–	–
SAM	–	11	2	–
RCM	–	–	9	–
HCM	–	33	24	–
Urea (mmol/l)	9.93 (8.27–11.50)	11.53 (8.80–13.79)	13.36 (10.90–18.19)	0.001^‡^
Creatinine (µmol/l)	132.9 (120.4–137.2)	147.5 (114.0–168.1)	151.8 (127.9–183.9)	0.032^‡^

Data are n or median (interquartile range [25th–75th percentile])

* Comparison between three groups (ACVIM B vs ACVIM C vs the control group)

† ACVIM C vs ACVIM B vs the control group (Kruskal–Wallis test)

‡ ACVIM C vs the control group (Quade’s non-parametric analysis of covariance [ANCOVA] with age as a covariate; reported *P* values adjusted for multiple comparisons were obtained directly from the statistical program [SPSS] output)

F = female; HCM = hypertrophic cardiomyopathy; M = male; RCM = restrictive cardiomyopathy; SAM = systolic anterior motion

Blood was collected either at admission or, in the case of cats in the ACVIM C group, as soon they were stable enough to tolerate the procedure. Among the cats with pulmonary oedema, seven received furosemide and two received angiotensin-converting enzyme (ACE) inhibitors before blood was collected. In cats with pleural effusion, 11 received furosemide, three received ACE inhibitors and one received pimobendan before blood collection. All three stable ACVIM C cats received furosemide before blood collection.

A total of 68 records of healthy cats were assessed during routine or surgical procedures; of them, 44 cats aged younger than 5 years were excluded, leaving 24 cats in the control group.

There were no significant differences in age and weight between the control, ACVIM B and ACVIM C groups (Table 1), and between the ACVIM C with pulmonary oedema and pleural effusion groups (Table 2).

**Table 2:** Signalment, complete blood count (CBC) parameters and CBC-derived inflammatory markers in cats with pulmonary oedema and cats with pleural effusion

Parameter	ACVIM C	ACVIM C	*P* value
	Pulmonary oedema	Pleural effusion	
Number of cats	14	16	–
Sex (M/F)	11/3	11/5	–
Age	13 years (8–16 years)	9 years (6 years and 3 months to 12 years)	0.343
Body weight (kg)	6.10 (4.28–8.65)	4.11 (3.50–6.48)	0.174
Urea (mmol/l)	16.28 ± 2.03	15.2 ± 1.62	0.678
Creatinine (µmol/l)	182.2 ± 13.2	146.4 ± 14.4	0.078
WBC (× 10^9^/l)	12.53 ± 5.45	11.98 ± 4.21	0.760
Neut (× 10^9^/l)	7.41 ± 5.17	8.88 ± 4.44	0.409
Mono (× 10^9^/l)	0.34 (0.23–0.54)	0.21 (0.17–0.50)	0.603
Lymph (× 10^9^/l)	2.16 ± 1.38	1.89 ± 1.13	0.570
NLR	2.40 (1.41–6.39)	4.68 (2.06–14.21)	0.198
MLR	0.200 (0.088–0.268)	0.200 (0.073–0.338)	0.851
SIRI (× 10^9^/l)	0.995 (0.380–3.035)	2.112 (0.428–3.312)	0.430

Data are n, mean ± SD or median (interquartile range [25th–75th percentile]). *P* values were calculated using the independent *t*-test for normally distributed data and the Mann–Whitney test for non-normally distributed data

ACVIM = American College of Veterinary Internal Medicine; F = female; Lymph = lymphocyte concentration; M = male; MLR = monocyte:lymphocyte ratio; Mono = monocyte concentration; Neut = neutrophil granulocyte concentration; NLR = neutrophil:lymphocyte ratio; SIRI = system inflammation response index; WBC = white blood cell count

Cats in ACVIM C had significantly higher serum creatinine and urea concentrations than the controls; however, this was not significantly different from cats in ACVIM B (Table 1).

No significant differences were observed in any of the investigated inflammatory and renal markers between cats with pulmonary oedema and those with pleural effusion (Table 2).

**Figure 1:**
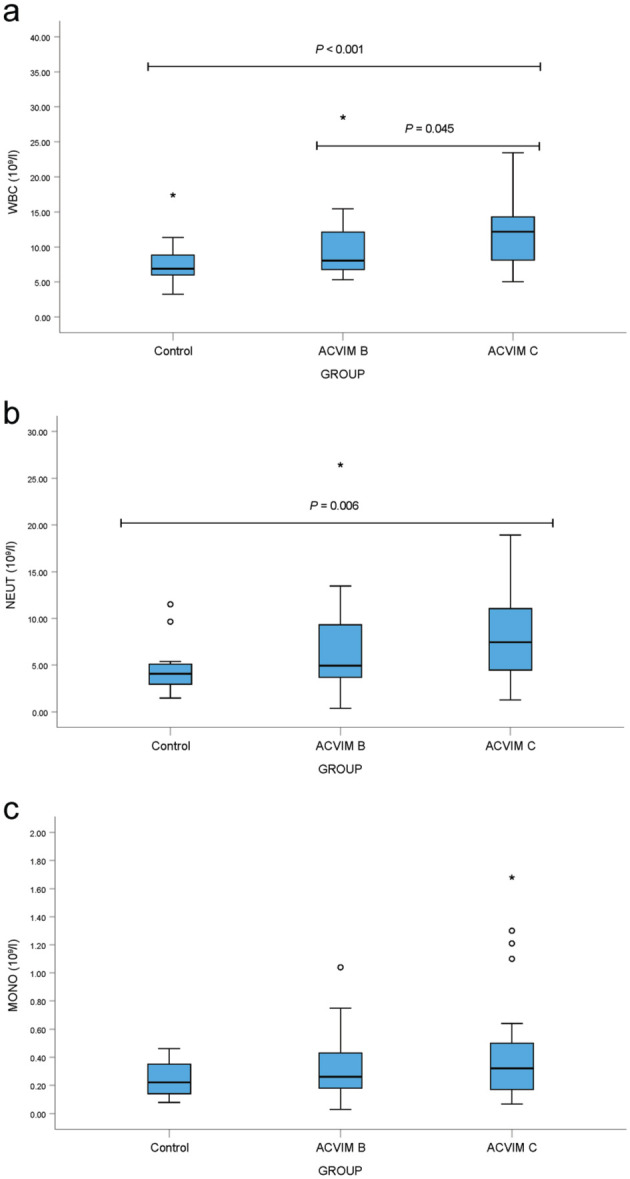
Box plots of complete blood count parameters: (a) white blood cell count (WBC), (b) neutrophil (NEUT) and (c) monocyte (MONO) concentrations in healthy cats and cats with cardiomyopathy (American College of Veterinary Internal Medicine [ACVIM] stages B and C). Boxes represent the interquartile range (IQR), with the horizontal line indicating the median. Whiskers extend to 1.5 × IQR. Circles denote outliers and asterisks indicate extreme outliers. Statistical comparisons were performed using Quade’s non-parametric analysis of covariance (ANCOVA); reported *P* values, adjusted for multiple group comparisons, were obtained directly from the statistical program (SPSS) output table

**Figure 2:**
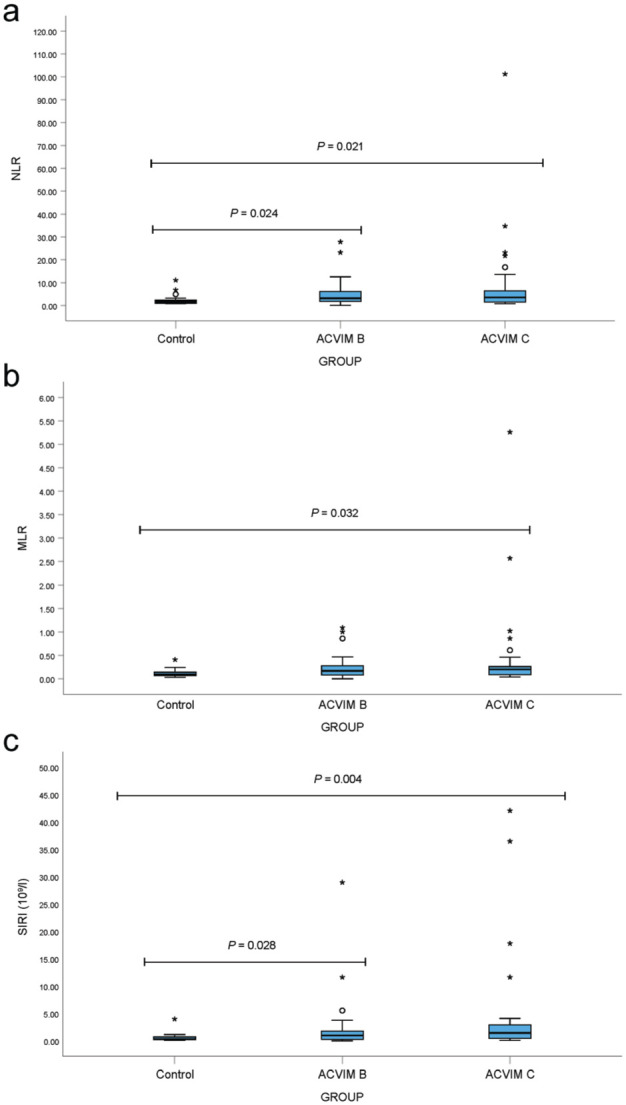
Box plots of complete blood count-derived inflammatory markers: (a) neutrophil:lymphocyte ratio (NLR), (b) monocyte:lymphocyte ratio (MLR) and (c) systemic inflammation response index (SIRI) in healthy cats and cats with cardiomyopathy (American College of Veterinary Internal Medicine [ACVIM] stages B and C). Boxes represent the interquartile range (IQR), with the horizontal line indicating the median. Whiskers extend to 1.5 × IQR. Circles denote outliers and asterisks indicate extreme outliers. Statistical comparisons were performed using Quade’s non-parametric analysis of covariance (ANCOVA); reported *P* values, adjusted for multiple group comparisons, were obtained directly from the statistical program (SPSS) output table

Cats in ACVIM C had significantly higher WBC and neutrophil concentrations (Figure 1), and significantly higher NLR, MLR and SIRI values (Figure 2) compared with healthy cats; however, these parameters did not differ significantly between the ACVIM B and C groups, except for WBC. Cats in ACVIM B had significantly higher NLR and SIRI values than healthy cats, although MLR values did not differ significantly between these groups (Figure 2).

Lymphocyte concentrations were lower in cats in ACVIM B (2.07 ± 1.25 × 10^9^/l) and C (2.09 ± 1.30 × 10^9^/l) compared with controls (2.39 ± 1.09 × 10^9^/l), but differences were not significant. Monocyte concentrations (Figure 1) also showed no significant group differences.

A post-hoc power analysis showed high power (>0.80^31^) for WBC (0.969), neutrophil concentration (0.802), SIRI (0.863) and urea concentration (0.907). Although NLR approached the threshold (0.769), a lower power was found for MLR (0.653), creatinine concentration (0.614), monocyte concentration (0.502) and lymphocyte concentration (0.107).

## Discussion

Cats in ACVIM stage C exhibited elevated haematological markers of inflammation compared with apparently healthy controls. Specifically, WBC concentrations were significantly higher in ACVIM C cats than in both stage B and control groups. In addition, NLR, MLR and SIRI values were significantly increased in the ACVIM C group compared with controls; however, no significant differences were observed between ACVIM B and C for these markers. Notably, cats in ACVIM B showed significantly higher NLR and SIRI values compared with healthy controls, while MLR did not differ between these two groups.

These findings may be attributed to a chronic inflammatory response in both ACVIM stage B and C cats. Previous studies reported that cats with CHF have significantly higher acute-phase proteins, such as leucine-rich alpha-2-glycoprotein 1, serum amyloid A (SAA) and ceruloplasmin, compared with healthy cats.^32^ However, little information is available on CBC-derived inflammatory markers in cats, and only one study has been published specifically on cats with heart disease, reporting a correlation of NLR with echocardiographic parameters and prognosis of feline HCM.^25^

In the present study, cats in ACVIM B and C had significantly higher NLR values than healthy cats. Similar results were reported in a previously published study in cats.^25^ However, unlike in our study, Fries et al^25^ reported a significant difference in NLR between cats in ACVIM B and C; this discrepancy may be attributed to differences in the included cat population. Apart from WBC, other examined markers (concentrations of neutrophils, monocytes and lymphocytes, NLR, MLR and SIRI) were not significantly different between ACVIM C and ACVIM B cats.

Increased WBC and neutrophil concentrations have been found in dogs with CHF,^16–18,20^ findings that align with the present study. Moreover, in dogs with MMVD and CHF, NLR,^21^ MLR and PLR^22^ were significantly increased^21,22^ and predicted a shorter survival time.^22^

Cats in ACVIM B and ACVIM C had significantly higher SIRI values than healthy cats. Although SII has been reported in various diseases in cats,^33,34^ the present study is the first to report SIRI, which may indicate a chronic type of inflammation in cats with cardiomyopathies. These markers have been reported as valuable in predicting CHF in human patients.^15,35^

In our study, cats in ACVIM C had significantly higher MLR values than healthy cats, a finding similar to that previously reported in dogs with MMVD.^23^ Monocytes are known to be involved in myocardial remodelling,^6^ which may explain the elevated SIRI and MLR observed in our cats with CHF. In human patients, increased peripheral inflammation, monocytosis and differentiation of monocytes into anti-inflammatory and profibrotic M2 macrophages have been associated with heart failure with preserved ejection fraction.^36^

Lymphopenia has been reported in feline cardiac disease,^37^ particularly in cases of cardiogenic pleural effusion. In our study, lymphopenia was equally represented in both the pleural effusion and pulmonary oedema groups, with no significant difference in lymphocyte concentrations between them. Similarly, when comparing lymphocyte concentrations to the control or ACVIM B groups, no significant differences were observed. It is important to note that CBC parameters mostly remained within the reference intervals, a finding also reported in dogs with CHF.^18,38^ The reference intervals used in CBC analyses are typically determined across a wide range of ages, breeds and sexes, which may not be entirely relevant for the examined age group. Therefore, we propose using age- and sex-matched control groups in studies.

Cats in ACVIM C had significantly increased serum urea and creatinine concentrations compared with controls. Increased renal values may reflect renal hypoperfusion, cardiorenal syndrome or the effects of therapy.^32,39,40^ Similarly, in dogs with MMVD, chronic kidney disease (CKD) stage has been found to correlate with MMVD stage.^41^ In our study, most cats in ACVIM B and C had mild azotaemia. In cats with early CKD, CBC-derived inflammatory markers did not significantly differ from healthy cats.^34^ Therefore, the differences in inflammatory markers between cats with CHF and healthy cats are likely associated with the inflammatory phenotype of CHF and cardiorenal syndrome rather than azotaemia per se.

We found no studies describing the degree of inflammation comparing pleural effusion and pulmonary oedema in either the human or veterinary literature.

Our results showed no significant differences in any of the examined parameters between cats with pulmonary oedema and cats with pleural effusion. Although the difference in SIRI between pleural effusion and pulmonary oedema groups was not statistically significant, median SIRI was over twice as high in the pleural effusion group (2.112 × 10^9^/l vs 0.995 × 10^9^/l), suggesting a potentially meaningful difference in inflammatory response that may become significant in larger samples. In human patients, SIRI is commonly associated with chronic inflammation and immune dysregulation, particularly in chronic diseases such as cardiovascular diseases and cancer.^14,42–44^

This study has some limitations, notably the small sample size. Although the post-hoc power analysis showed sufficient power for key variables (WBC, neutrophil and urea concentrations, SIRI) and near-threshold power for NLR, it was lower for MLR, monocyte, lymphocyte and creatinine concentrations. Thus, although some comparisons are reliable, non-significant findings for low-power variables should be interpreted with caution.

Despite statistical control for age, the younger median age in the control group may still pose residual confounding.

Body and muscle condition scores were not recorded, although low muscle condition score may affect serum creatinine.^45,46^ In addition, control cats did not undergo echocardiography; therefore, subclinical cardiomyopathy cannot be excluded.

Furthermore, in some cats with pleural effusion, pulmonary parenchyma could not be assessed because of the superimposition of pleural fluid on the lung parenchyma and/or atelectasis of the lung parenchyma due to compression. In these cases, mild concurrent pulmonary oedema may have remained undiagnosed.

Another limitation of the study is the fact that, despite predominantly including cats with HCM, some with RCM phenotype were included as well, which may have affected the results. However, the aim of our study was to evaluate the extent of inflammation in cardiomyopathies in general. Cardiomyopathy phenotypes vary, and it is possible that one phenotype progresses into another.^47^ In humans, HCM and primary RCM have a similar genetic background as they are both caused mainly by variants in sarcomeric genes.^48^ Furthermore, several inflammatory biomarkers – such as SAA, CRP, alpha-1-acid glycoprotein, leucine-rich alpha-2-glycoprotein 1, ceruloplasmin, N-terminal pro-B-type natriuretic peptide, cardiac troponin I and haptoglobin – have been investigated in cats with cardiomyopathies.^32^ The same study found that cardiomyopathy phenotype did not affect the concentration of any investigated biomarker.^32^

As a result of the retrospective nature of the study, blood smears were not available for assessing platelet clumps or band neutrophils. Therefore, platelet (PLT) and inflammatory markers including PLT data (PLR and SII) are not included in this article because they could not be reliably assessed. Leukograms may have been influenced by hospitalisation and illness severity. Without blood smear analysis, distinguishing stress from inflammation was limited. Stress due to dyspnoea and inflammation may have coexisted in diseased cats, and chronic stress may have had an impact on the CBC-derived inflammatory markers. However, during clinic visits, both the HCM and control cats were accompanied by their owners during all procedures, which reduced the possibility of these being stressful events for the animals. The environmental conditions for both HCM and control cats were similar, and control cats did not show stress leukograms. Furthermore, there is evidence that chronic inflammation is already present in cats with mild preclinical HCM,^8,32^ and there is a possibility that chronic stress is just another component of chronic inflammation. Therefore, the consequence may be no observed difference between the ACVIM groups based on the parameters investigated.

Furthermore, because of the retrospective nature of the study, not all potential causes of systemic inflammation could be completely excluded.

Variations in hydration status may have influenced blood cell ratios. Additional standard inflammatory markers like SAA and albumin:globulin ratio (AGR) were not assessed, although prior studies^33,49^ have shown correlations between these and CBC-derived inflammatory markers in cats, as well as with clinical indicators of HCM.^25^ Further research is needed to define cutoff values of CBC-derived inflammatory markers and explore associations with SAA and AGR.

## Conclusions

Our results suggest that CBC parameters and CBC-derived inflammatory markers can provide evidence of chronic inflammation in feline cardiomyopathies. Furthermore, CBC-derived inflammatory markers (NLR, MLR and SIRI) were significantly higher in cats with ACVIM C compared with healthy cats, indicating a pronounced chronic inflammatory state in CHF. In addition, cats in ACVIM B had significantly higher NLR and SIRI values compared with healthy cats, indicating the presence of inflammation in the preclinical phase. There was no significant difference in the extent and type of inflammation between pleural effusion and pulmonary oedema.
